# Supplementary material for: Polyketide synthase gene clusters in the ericoid mycorrhizal fungus Oidiodendron maius: genome-wide prediction, expression profiling, and functional characterization of knockout mutants
Source: Front Plant Sci. 2026 Sep 16;17:1924430. doi: 10.3389/fpls.2026.1924430 (PMC13623903; doi:10.3389/fpls.2026.1924430)
Supplement: Supplementary file 1 [file SupplementaryFile1.zip › Supplementary Materials/Supplementary Folder 1.zip!/Oidma1_AssemblyScaffolds_Repeatmasked/knownclusterblast/cluster29/jgi.p_Oidma1_32113_mibig_hits.html]

| MIBiG Protein | Description | MIBiG Cluster | MiBiG Product | % ID | % Coverage | BLAST Score | E-value |
| --- | --- | --- | --- | --- | --- | --- | --- |
| ABP53487.1 | Methyltransferase\_type\_11 | BGC0001041 | Salinosporamide\_biosynthetic\_gene\_cluster | 46 | 93.536121673 | 196 | 4.8e-50 |
| AAS79475.1 | putative\_merR-family\_transcriptional\_regulator | BGC0000035 | Chalcomycin\_biosynthetic\_gene\_cluster | 44 | 87.8326996198 | 194 | 1.8e-49 |
| ABR67773.1 | unknown | BGC0000316 | Capreomycin\_biosynthetic\_gene\_cluster | 39 | 94.2965779468 | 170 | 2.1e-42 |
